# Fermented Liquid Feeding Modulates Intramuscular Fat Deposition and Lipid Composition in Pork: Integrated Metabolomic and Proteomic Insights

**DOI:** 10.3390/foods15152725

**Published:** 2026-08-03

**Authors:** Li Wang, Hefeng Luo, Zhongyang Guo, Tianle Gao, Rui Zhou, Liang Hu, Hong Chen, Yong Zhuo, Bin Feng, Lianqiang Che, De Wu, Zhengfeng Fang

**Affiliations:** 1 Key Laboratory for Animal Disease-Resistance Nutrition of China Ministry of Education, Institute of Animal Nutrition, Sichuan Agricultural University, Chengdu 611130, China; wangli202401@gmail.com (L.W.);; 2 Sichuan Dekon Livestock Foodstuff Group, Chengdu 610200, China; 3 College of Food Science, Sichuan Agricultural University, Yaan 625014, China

**Keywords:** fermented liquid feeding, intramuscular fat, pork quality, lipid composition, metabolomics

## Abstract

Fermented liquid feeding (FLF) has been proposed as a nutritional intervention with the potential to influence pork quality, yet its effects on intramuscular fat deposition and muscle lipid composition remain unclear. In this study, pigs were fed a basal dry feed, liquid feed, or FLF regimen for 173 days, and porcine muscle samples were subsequently evaluated for carcass traits, meat quality, lipid composition, and molecular changes associated with lipid deposition. Compared with CON, FLF reduced average backfat thickness, while intramuscular fat and triglyceride contents in the longissimus thoracis muscle were greater than those in CON and LF (*p* < 0.05). Fatty-acid profiling showed that FLF increased several major fatty acids, including palmitic acid. Metabolomic analysis identified contrast-specific changes in phosphatidylserine and phosphatidylethanolamine species. Proteomic analysis, together with Western blotting and RT-qPCR, showed higher abundances of PPARγ, FASN, and downstream lipogenic transcripts in the FLF group. These findings demonstrate that FLF modulates lipid deposition and lipid composition in porcine muscle and provide mechanistic insight into the nutritional regulation of pork quality formation.

## 1. Introduction

Pork represents a major source of animal protein in human diets worldwide. Nevertheless, long-term genetic selection strategies primarily targeting rapid growth and increased lean meat yield have been associated with a gradual deterioration in pork quality attributes [1]. Dietary intervention has therefore emerged as a practical and effective approach to modulating pork quality, particularly through the optimization of feed ingredients and dietary formulation strategies.

Fermented feed, generated through controlled microbial fermentation processes, can substantially reduce antinutritional components commonly present in cereal-based diets. Previous studies have demonstrated that fermented feed supplementation contributes to improved growth performance, enhanced immune responses, and better intestinal health in pigs [2]. In addition, the inclusion of fermented feed at levels ranging from 8% to 15% has been reported to positively influence pork quality traits, such as meat color, flavor characteristics, intramuscular fat (IMF) deposition, and water-holding capacity [3].

Fermented liquid feeding (FLF) is a specific form of fermented feed produced by mixing feed with water at ratios typically between 1:1.5 and 1:4, followed by fermentation until a stable microbial ecosystem is established [4]. Compared with fermented solid feed, the higher moisture content in FLF facilitates microbial fermentation and may preserve more probiotics and beneficial metabolites [4]. In addition, FLF shares some advantages with liquid feed (LF), such as promoting feed intake, improving feed digestibility, and reducing feed waste [4]. Previous studies have shown that FLF improves pig production performance and gut health [5,6,7]. However, reports on its impact on pork quality remain limited. A recent study reported that FLF increased IMF deposition in pigs [7], but the biological basis underlying this response remains unclear.

Metabolomic analysis enables comprehensive profiling of small-molecule metabolites in pork and provides insight into metabolic alterations, whereas proteomic approaches offer complementary information on the biological pathways and molecular processes associated with these changes [8]. In the field of meat science, combined proteomic and metabolomic strategies have increasingly been employed to elucidate the regulatory networks underlying meat quality traits [9].

In the present study, an integrated multi-omics framework incorporating proteomics, metabolomics, and targeted molecular measurements was applied to evaluate the effects of FLF on carcass traits, meat quality, and IMF deposition in finishing pigs. Particular emphasis was placed on exploring molecular changes associated with the FLF-related response in muscle lipid deposition.

## 2. Materials and Methods

### 2.1. Ethics Statement

Animal procedures followed the welfare requirements of Sichuan Agricultural University and were authorized by its Institutional Animal Care and Advisory Committee (protocol YYS20231201; approval date, 1 December 2023).

### 2.2. Animals, Diets, and Experimental Design

A total of 768 Duroc × Landrace × Yorkshire pigs, aged 23 days and weighing 6.88 ± 0.20 kg, were assigned according to sex and initial body weight to three treatments for 173 days. Each treatment included eight pens with 32 pigs per pen. The CON group was offered the basal diet in dry form, whereas the LF group received the basal diet mixed with water at 1:3 (*w*/*w*). The FLF diet contained 70% unfermented liquid basal diet and 30% fermented liquid basal diet (Table 1).

The fermented fraction was prepared by mixing the basal diet with water at approximately 35 °C at 1:3 (*w*/*w*). A microbial-enzyme preparation was added at 2 g/kg feed, and the mixture was mixed for 10 min and fermented anaerobically at 35 °C for 24 h. The preparation supplied *Lactobacillus plantarum* CICC 21790 (1 × 10^11^ CFU/g), xylanase (500 U/g), cellulase (500 U/g), and acid protease (5000 U/g). The final FLF diet was delivered using a WEDA liquid feeding system. Feed and water were available ad libitum. The pen was considered the experimental unit for growth performance measurements.

**Table 1:** Ingredient composition, analyzed chemical composition, and calculated nutrient levels of basal diets.

Ingredient	Phase (kg)					
	6–9	9–15	15–26	26–60	60–90	90–130
Ingredient (%)						
Corn (Grade 1)	26.50	32.20	-	-	-	-
Whey Powder (80%)	10.00	5.00	-	-	-	-
Broken Rice	10.00	10.00	-	-	-	-
Coconut Meal Powder	5.00	2.50	-	-	-	-
Cottonseed Enzymatic Protein	2.00	-	-	-	-	-
Glucose	2.50	2.50	-	-	-	-
Yeast Hydrolysate	2.50	1.25	-	-	-	-
Brown Rice Mixture	-	-	35.00	40.00	40.00	40.00
Wheat Flour (1.5)	10.00	15.00	8.90	3.00	3.00	3.00
Rice Polishing Bran	-	-	10.00	8.60	10.00	12.00
Wheat Germ	-	-	10.00	8.90	2.80	-
Soybean Meal (45%)	7.55	14.30	6.32	2.73	1.00	-
Rice Bran Meal	-	-	7.00	9.40	3.80	4.26
Expanded Soybean	8.00	5.00	5.00	-	-	-
Corn (Grade 2)	-	-	11.15	8.38	12.72	14.40
Fermented Soybean Meal	6.00	3.00	2.00	-	-	-
Limestone	-	-	1.17	1.28	1.20	1.10
Dicalcium Phosphate Type III	-	-	0.81	0.46	0.35	0.25
L-Lysine Sulfate (70%)	-	-	0.77	1.04	1.01	0.92
NaCl	-	-	0.35	0.35	0.35	0.35
Soybean Oil	1.95	1.75	0.30	-	-	-
L-Threonine (98.5%)	-	-	0.25	0.17	0.14	0.18
DL-Methionine (98.5%)	-	-	0.22	0.17	0.13	0.09
Betaine (96%)	-	-	0.05	-	-	-
Choline Chloride (60%)	-	-	0.05	-	-	-
L-Tryptophan (98%)	-	-	0.06	0.08	0.08	0.06
Valine (98%)	-	-	-	0.09	0.07	0.04
Corn DDGS	-	-	-	12.00	15.00	15.00
Rice Bran	-	-	-	-	5.00	5.00
Corn Germ Meal	-	-	-	3.00	3.00	3.00
Vitamin Premix-1	4.00	4.00	0.30	-	-	-
Mineral Premix-1	4.00	3.50	0.30	-	-	-
Mineral premix-2	-	-	-	0.20	0.20	0.20
Vitamin premix-2	-	-	-	0.15	0.15	0.15
Total	100.00	100.00	100.00	100.00	100.00	100.00
Analyzed nutrient composition						
Moisture	10.36	11.65	13.00	13.36	13.57	13.72
Crude protein, %	19.49	18.51	17.51	15.00	13.98	13.00
Crude fiber, %	2.49	2.07	2.24	3.31	2.92	2.96
Ether extract, %	6.68	5.53	3.52	2.96	2.96	2.85
Calcium, %	0.60	0.60	0.65	0.60	0.55	0.50
Total phosphorus, %	0.61	0.57	0.52	0.56	0.51	0.49
Calculated nutrient levels						
Digestible energy (kcal/kg)	3427	3397	3372	3350	3300	3250
SID lysine, %	1.35	1.25	1.15	1.05	0.95	0.85
SID Methionine, %	0.56	0.51	0.45	0.39	0.35	0.30
SID threonine, %	0.83	0.78	0.72	0.65	0.59	0.53
SID tryptophan, %	0.27	0.25	0.23	0.21	0.19	0.17

Per kilogram of complete diet, vitamin premix 1 supplied vitamin A, 10,000 IU; vitamin D_3_, 2500 IU; vitamin E, 40 IU; vitamin K_3_, 3.0 mg; thiamine, 1.5 mg; riboflavin, 4.0 mg; pyridoxine, 2.0 mg; vitamin B_12_, 20 μg; niacin, 20 mg; calcium pantothenate, 12 mg; folic acid, 0.8 mg; and biotin, 0.10 mg. Mineral premix 1 supplied, per kilogram of complete diet, Fe, 120 mg; Cu, 20 mg; Zn, 120 mg; Mn, 40 mg; I, 0.50 mg; and Se, 0.30 mg. Per kilogram of complete diet, vitamin premix 2 supplied vitamin A, 8000 IU; vitamin D_3_, 1600 IU; vitamin E, 30 IU; vitamin K_3_, 2.0 mg; thiamine, 1.0 mg; riboflavin, 3.0 mg; pyridoxine, 1.2 mg; vitamin B_12_, 15 μg; niacin, 12 mg; calcium pantothenate, 8.0 mg; folic acid, 0.40 mg; and biotin, 0.05 mg. Mineral premix 2 supplied, per kilogram of complete diet, Fe, 100 mg; Cu, 15 mg; Zn, 100 mg; Mn, 30 mg; I, 0.40 mg; and Se, 0.30 mg. Digestible energy and standardized ileal digestible amino-acid levels were calculated, whereas moisture, crude protein, crude fiber, ether extract, calcium, and total phosphorus were measured analytically.

### 2.3. Carcass Traits and Sample Collection

After feed withdrawal for 12 h, one representative pig from each pen entered a 4 h lairage period with water available. Electrical stunning followed by exsanguination was used for humane slaughter, after which the carcasses were chilled at 4 °C. Dressing percentage was calculated from the recorded carcass and preslaughter weights. Backfat depth was measured at the first rib, last rib, and final lumbar vertebra and averaged, and eye muscle area (EMA) was determined on the 13th-rib cross-section.

Sections of the longissimus thoracis (LT) were assigned prospectively to each analysis. The portion spanning the first to second thoracic vertebrae anterior to the last rib was used for 45 min pH and color; the third to fourth vertebrae were used for drip and cooking loss; and the seventh to ninth vertebrae were used for shear force. Meat-quality samples were sealed separately and transported at 4 °C. Right-side LTs collected for molecular and biochemical assays were frozen in liquid nitrogen and kept at −80 °C. Approximately 200 g from the left-side 10th-rib region was stored at −20 °C for fatty-acid analysis.

### 2.4. Meat Quality and Physicochemical Analyses

Procedures were adapted from Huang et al. [10]. Muscle pH was recorded at 45 min, 24 h, and 48 h postmortem with a pH-STAR meter (SFK-Technology, Herlev, Denmark) after calibration with pH 4.0 and 7.0 buffers. Color coordinates (L*, a*, and b*) were obtained at the same time points with a Minolta CR-300 chromameter (Konica Minolta, Tokyo, Japan) after a 30 min bloom at 4 °C. Measurements used D65 illumination, a 10° observer, and an 8 mm aperture; three readings were averaged for each pig.

Drip loss was measured with a suspension-bag procedure. An approximately 40 g LT strip cut parallel to the fibers was weighed (W1), hung freely inside a sealed, inflated polyethylene bag, and held at 4 °C without contacting the bag surface. At 24 and 48 h, the sample was removed, blotted by the same trained operator, and reweighed (W2); drip loss was calculated as [(W1 − W2)/W1] × 100. For cooking loss, approximately 120 g of LT was weighed and steamed, without a bag, for 30 min in a single batch over boiling water. After 20 min of suspended cooling, the surface was blotted and the final weight recorded; cooking loss was calculated using the same equation.

Cooked samples were refrigerated at 4 °C for 24 h before shear testing. Six cores (1.27 cm diameter) were removed along the fiber axis while avoiding visible fat and connective tissue. Each core was cut once across the fibers on a TA.XT Plus texture analyzer (Stable Micro Systems, Surrey, UK) fitted with a 60° knife blade (1.016 mm thick), operated in compression mode at 200 mm/min with a 2-N preload. The six peak-force values were averaged for each pig. Three trained assessors assigned marbling scores according to NPPC standards.

### 2.5. Muscle Chemical Composition

Fresh LT tissue (40 g) was sliced and freeze-dried at −50 °C for 48 h in a Labconco unit (Kansas City, MO, USA) until its weight stabilized [11]. Moisture was derived from the change in sample weight. The dried material was then analyzed for crude protein and IMF using AOAC-approved procedures.

### 2.6. Muscle Amino Acid Composition

For each sample, 0.15 g of LT was digested in 13 mL of 6 M hydrochloric acid at 110 °C for 24 h. The hydrolysate was diluted to 100 mL with ultrapure water and filtered through a 13 m hydrophilic polyethersulfone membrane (0.45 μm; Jinteng, Tianjin, China). An aliquot was vacuum-dried at 60 °C to remove residual acid, redissolved in sodium citrate buffer, and analyzed on an S-433D automatic amino-acid analyzer (Sykam, Eresing, Germany).

### 2.7. Fatty Acid Composition

Fresh-muscle lipids were isolated by the Folch procedure [12]. Before the extraction solvent was added, 1.00 mL of triundecanoin in methanol (4.00 mg/mL; 4.00 mg per sample) was pipetted onto each weighed sample. The extract was saponified in 2% (*w*/*v*) potassium hydroxide in methanol at 70–80 °C for 20–30 min until visible oil droplets disappeared, then methylated at 80 °C for 10 min. Fatty-acid methyl esters were separated on a GC-2014 gas chromatograph (Shimadzu, Kyoto, Japan) and identified against authentic retention-time standards. Concentrations were calculated relative to triundecanoin, with response and molecular-weight corrections, and reported as mg/100 g fresh muscle.

### 2.8. Biochemical Parameters

LT tissue was homogenized with 0.9% saline at 1:10 (*w*/*v*) and centrifuged at 2500× *g* for 15 min at 4 °C. Commercial kits (Nanjing Jiancheng Biologicals, Nanjing, China) were used to determine TG, total cholesterol (T-CHO), LDL cholesterol, and HDL cholesterol in the supernatant, with absorbance read on a SpectraMax M2 microplate reader (Molecular Devices, San Jose, CA, USA).

### 2.9. Western Blot Analysis

Western blotting followed Huang et al. [13] under the conditions specified below. LT samples were extracted in RIPA buffer, and protein concentration was measured with a BCA kit (Thermo Fisher Scientific, Waltham, MA, USA). Aliquots containing 20 μg of protein were resolved by SDS-PAGE, transferred to polyvinylidene fluoride membranes, and blocked with 5% bovine serum albumin.

Membranes were incubated overnight at 4 °C with rabbit anti-PPARγ (Zen BioScience, Chengdu, China; 340844; 1:1000), rabbit anti-FASN (Zen BioScience; R381582; 1:1000), or mouse anti-GAPDH (Servicebio, Wuhan, China; GB12002; 1:3000). The appropriate HRP-conjugated goat anti-rabbit (GB23303; 1:3000) or goat anti-mouse IgG (GB23301; 1:3000; Servicebio) was applied for 1 h at room temperature. Bands were developed by enhanced chemiluminescence, imaged with a ChemiDoc system (Bio-Rad, Hercules, CA, USA), and quantified in Image Lab (version 6.1). PPARγ and FASN were normalized to GAPDH. Four biological samples per treatment were selected before analysis so that the 12 samples could be run on one gel and membrane, thereby avoiding inter-blot variation.

### 2.10. Real-Time Quantitative Polymerase Chain Reaction (RT-qPCR)

Total RNA was extracted from LT with RNAiso Plus (TaKaRa, Dalian, China), and concentration and purity were checked spectrophotometrically. Equal RNA inputs were converted to cDNA with the PrimeScript RT reagent kit (TaKaRa). SYBR Select Master Mix and a QuantStudio 6 Flex instrument (Applied Biosystems, Foster City, CA, USA) were used for amplification. Six biological replicates were analyzed per treatment, with three technical reactions for each sample. Each assay produced a single melting peak. Amplification efficiency was obtained from five-point standard curves prepared with serially diluted pooled cDNA and calculated as E (%) = [10^−1/slope^ − 1] × 100. Efficiencies for GAPDH, SCD, DGAT1, and PLIN2 were 98.7%, 96.4%, 94.9%, and 97.2%, with R^2^ values of 0.998, 0.997, 0.996, and 0.999, respectively. Table 2 lists the primer sequences. Relative transcript abundance was calculated using the 2^−ΔΔCt^ method using GAPDH as the reference. Raw GAPDH Ct values did not differ among treatments and their overall coefficient of variation was 0.258%.

### 2.11. Untargeted Metabolomics Analysis

Muscle tissue (approximately 100 mg) was extracted in chilled methanol containing internal standards. After vortexing, samples were centrifuged at 14,000× *g* for 20 min at 4 °C and the recovered supernatant was vacuum-dried. The residue was dissolved in 50 μL of methanol/water/formic acid (66.6:33.3:0.1, *v*/*v*/*v*), mixed, and clarified by centrifugation before injection.

Chromatography was performed on an ACQUITY UPLC system (Waters, Milford, MA, USA) equipped with a T3 C18 column (2.1 × 100 mm, 1.8 μm; 40 °C) and coupled to an Orbitrap Exploris 480 mass spectrometer (Thermo Fisher Scientific). Water and acetonitrile, each containing 0.1% formic acid, served as the mobile phases at 0.3 mL/min. Spectra were collected in positive and negative ion modes. Compound Discoverer 3.3 was used for annotation against mzCloud, ChemSpider, HMDB, and LIPID MAPS. Pooled quality-control samples, prepared from equal aliquots of every extract, were interspersed through the sequence to assess analytical stability. PCA and OPLS-DA were applied. R^2^X, R^2^Y, and Q^2^ described model fit, and 200 permutations were used to assess overfitting. Differential metabolites were defined by VIP > 1 and *p* < 0.05 (Student’s *t*-test). The PS and PE heatmaps contain the species that met these criteria in the specified pairwise contrast and do not imply the significance of both lipid classes in both contrasts.

### 2.12. Proteomic Analysis

Following homogenization in lysis buffer, samples were centrifuged and the protein concentration of the extracts was determined using the Bradford assay. Proteins were recovered via methanol-chloroform precipitation, reduced with dithiothreitol, alkylated with chloroacetamide, and digested with trypsin (1:100, *w*/*w*) for 14–16 h at 37 °C. The resulting peptides were cleaned on SEP-PAK C18 cartridges.

Peptides were analyzed on an EASY-nLC 1200 coupled to an Orbitrap Exploris 480 (Thermo Fisher Scientific, Waltham, MA, USA). An Acclaim PepMap 100 C18 column (75 μm × 20 mm, 3 μm) was used with a 52 min linear gradient from 3% to 35% acetonitrile, and spectra were acquired in data-dependent mode.

Proteome Discoverer 3.1 was used for protein identification and label-free quantification against the UniProtKB *Sus scrofa* reference proteome. False discovery rates at both the peptide and protein levels were maintained below 1%.

### 2.13. Statistical Analysis

Statistical analyses were conducted in SPSS 22.0 (SPSS Inc., Chicago, IL, USA). The pen was the experimental unit for growth performance. For post-slaughter endpoints, the pig selected from each independent pen represented that pen and served as the unit for carcass, meat-quality, chemical, amino-acid, fatty-acid, omics, and molecular measurements. Dietary treatment was modeled as a fixed effect. When the overall treatment test was significant, Duncan’s multiple-range procedure was used to compare means. Values are reported as means ± SEM; *p* < 0.05 denoted significance and 0.05 ≤ *p* < 0.10 denoted a tendency.

Omics variables were log_2_-transformed and the z-score was standardized when appropriate. Metabolites were considered differential at VIP > 1 and *p* < 0.05 by Student’s *t*-test. Proteins were classified as differentially abundant when the FDR-adjusted *p* value was <0.05 and the fold change was ≥1.20 or ≤0.83 (|log_2_ fold change| ≥ 0.263). Positive log_2_ fold changes denote higher abundance in the first group of a stated comparison. KEGG and SMPDB were used for pathway enrichment, and Cytoscape (version 3.9.1) was used to display protein-protein interactions.

## 3. Results

### 3.1. Carcass Traits

Table 3 summarizes the carcass outcomes. Preslaughter live weight and carcass weight were higher in FLF than in CON or LF (*p* < 0.05). Last-rib and average backfat thickness were lower in FLF than in CON (*p* < 0.05), whereas LF did not differ from either group for these measurements.

### 3.2. Meat Quality Attributes

Meat quality parameters are presented in Table 4. Postmortem pH values, color parameters (L*, a*, and b*), drip loss, and cooking loss were not significantly affected by LF or FLF supplementation (*p* > 0.05). Although no statistical difference was detected, pigs fed the FLF diet tended to have lower shear force values than those in the other groups (0.05 < *p* < 0.10).

### 3.3. Chemical Composition, Amino Acid Profile, and Fatty Acid Profile in LT Muscle

The chemical composition of the longissimus thoracis muscle is shown in Table 5. Compared with the CON and LF groups, FLF increased IMF content and TG concentration (*p* < 0.05). Moisture, crude protein, T-CHO, HDL-C, and LDL-C concentrations were not affected by dietary treatment (*p* > 0.05).

Analysis of muscle amino-acid composition (Table 6) indicated that FLF increased essential amino acids (EAA), lysine, and leucine relative to CON (*p* < 0.05). Both LF and FLF increased alanine, phenylalanine, and serine compared with CON (*p* < 0.05).

As shown in Table 7, the FLF regimen increased palmitic acid (C16:0) in LT muscle compared with CON and LF (*p* < 0.05). The concentrations of C18:0, C18:1n9, and C18:2n6, as well as total SFA, MUFA, PUFA, and n-6 PUFA, were also greater in the FLF group (*p* < 0.05).

### 3.4. Metabolomic Changes Associated with FLF in LT Muscle

**Figure 1:**
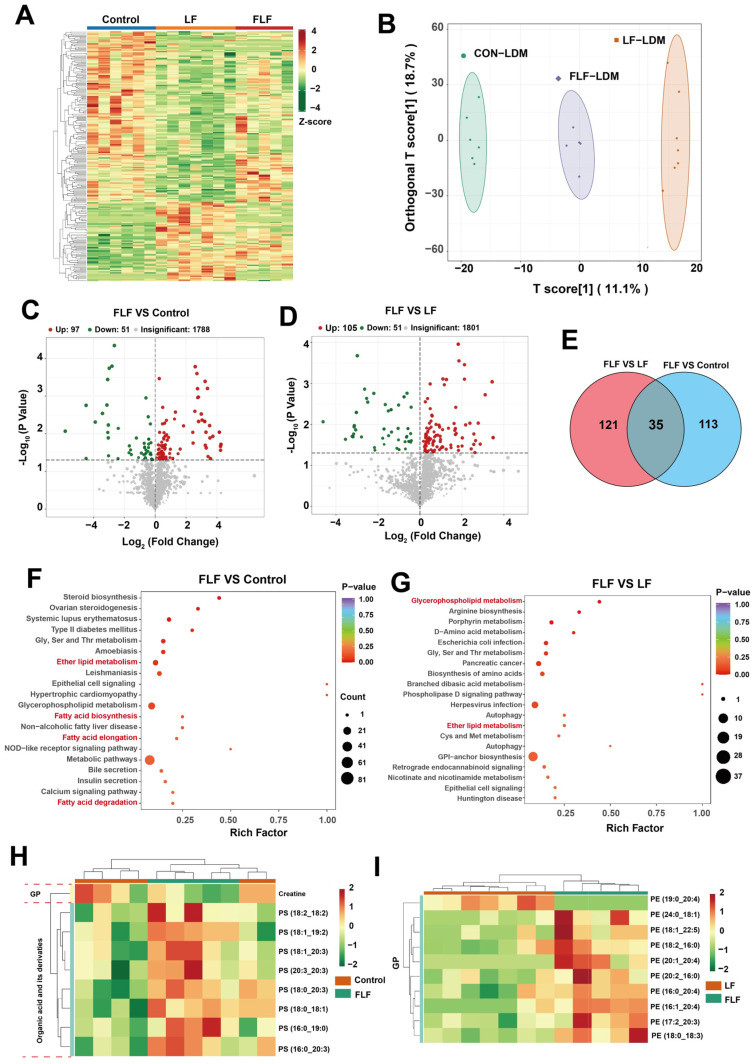
Metabolomic analysis of LT muscle. (**A**) Heatmap of differential metabolites. (**B**) OPLS-DA score plot. (**C**,**D**) Volcano plots for FLF versus CON (**C**) and FLF versus LF (**D**). (**E**) Venn diagram of metabolites differing in the FLF-versus-LF and FLF-versus-CON comparisons. (**F**,**G**) KEGG enrichment analyses for FLF versus CON (**F**) and FLF versus LF (**G**). (**H**) Heatmap of the nine PS species meeting VIP > 1 and *p* < 0.05 in the FLF-versus-CON comparison. (**I**) Heatmap of the nine PE species meeting the same criteria in the FLF-versus-LF comparison. The heatmaps display all PS or PE species meeting the predefined criteria in the relevant comparison. All metabolomic analyses were performed with six biological replicates per group.

Because FLF increased IMF content, metabolomic analysis was performed to characterize the associated metabolic changes in LT muscle. Figure 1A shows the overall distribution of differential metabolites. The OPLS-DA score plot showed separation among the three dietary groups (Figure 1B). The model exhibited strong explanatory and predictive performance (R^2^X = 0.471, R^2^Y = 0.999, and Q^2^ = 0.707), and a 200-permutation test indicated that it was not overfitted (Table S1). Relative to CON, FLF was associated with 148 differential metabolites, including 97 with higher abundance and 51 with lower abundance (Figure 1C). Relative to LF, FLF was associated with 156 differential metabolites, including 105 with higher abundance and 51 with lower abundance (Figure 1D). Thirty-five metabolites were shared between the FLF-versus-CON and FLF-versus-LF comparisons (Figure 1E). KEGG enrichment showed that the FLF-versus-CON comparison involved ether-lipid metabolism, fatty-acid biosynthesis, fatty-acid elongation, and fatty-acid degradation (Figure 1F), whereas the FLF-versus-LF comparison involved glycerophospholipid and ether-lipid metabolism (Figure 1G). Eight PS species met the predefined criteria of VIP > 1 and *p* < 0.05 in the FLF-versus-CON comparison and are displayed in Figure 1H. Ten PE species met the same criteria in the FLF-versus-LF comparison and are displayed in Figure 1I.

### 3.5. Proteomic Changes Associated with the Lipid-Deposition Response to FLF

**Figure 2:**
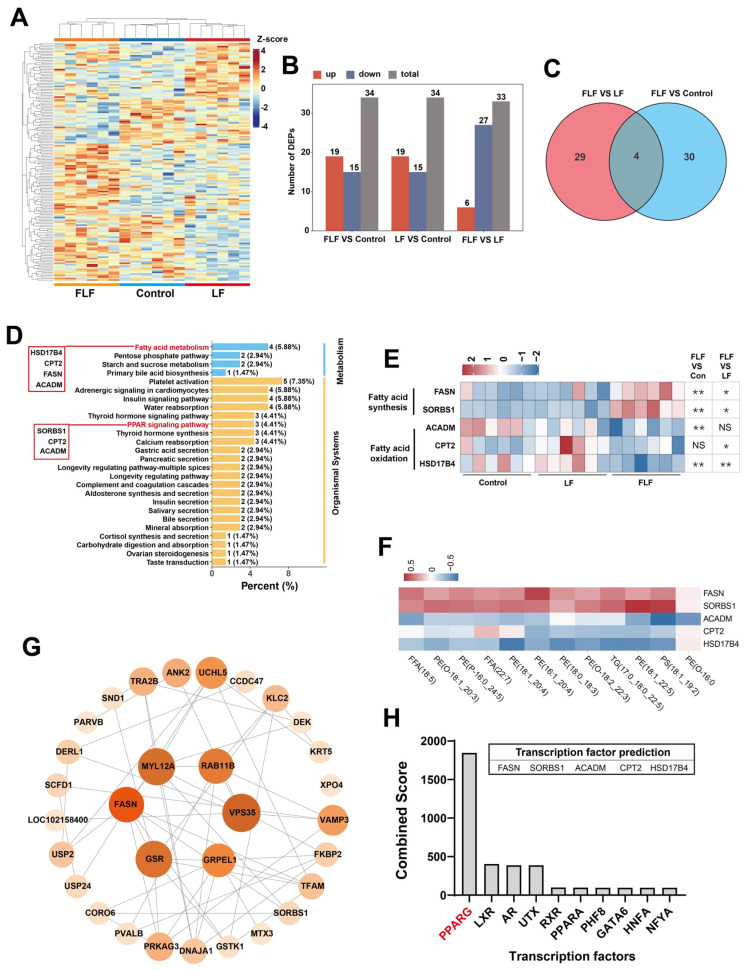
Proteomic analysis of LT muscle. (**A**) Heatmap of differentially abundant proteins. (**B**) Numbers of proteins with higher and lower abundance in FLF versus CON, LF versus CON, and FLF versus LF. (**C**) Venn diagram of proteins differing in the FLF-versus-LF and FLF-versus-CON comparisons. (**D**) KEGG pathway enrichment analysis. (**E**) Heatmap of proteins related to fatty-acid metabolism; the comparison labels indicate FLF versus CON and FLF versus LF. * *p* < 0.05; ** *p* < 0.01; NS, not significant. (**F**) Correlation heatmap between PS/PE species and proteins related to fatty-acid metabolism. (**G**) Protein-protein interaction network; node darkness and size are proportional to betweenness centrality. (**H**) Transcription-factor enrichment prediction performed using Enrichr; the top 10 enriched transcription factors are shown. All proteomic analyses were performed with six biological replicates per group.

Proteomic analysis was performed to characterize protein abundance changes associated with the lipid-deposition response observed in the FLF group. Figure 2A shows the overall abundance patterns in LT muscle. Using a positive log_2_ fold change to indicate higher abundance in the first group named, 19 proteins showed higher abundance and 15 showed lower abundance in FLF versus CON; 19 proteins showed higher abundance and 15 showed lower abundance in LF versus CON; and 6 proteins showed higher abundance and 27 showed lower abundance in FLF versus LF (Figure 2B). Four differential proteins were shared between the FLF-versus-CON and FLF-versus-LF comparisons (Figure 2C). KEGG and GO analyses indicated enrichment of lipid metabolism related processes, including the PPAR signaling pathway (Figure 2D). FASN and SORBS1 showed higher abundance in FLF than in both CON and LF. ACADM showed lower abundance in FLF than in CON but did not differ from LF; CPT2 showed lower abundance in FLF than in LF but did not differ from CON; and HSD17B4 showed lower abundance in FLF than in both comparison groups (Figure 2E). Lipid metabolites were generally positively correlated with FASN and SORBS1 and negatively correlated with ACADM, CPT2, and HSD17B4 (Figure 2F). FASN was a central node in the PPI network (Figure 2G), and transcription-factor enrichment identified PPARγ as a candidate regulator (Figure 2H). Collectively, these analyses identify the PPARγ-FASN axis as a candidate pathway associated with the response to the FLF regimen.

### 3.6. FLF Is Associated with Increased Abundance of PPARγ-FASN Axis Components in LT Muscle

**Figure 3:**
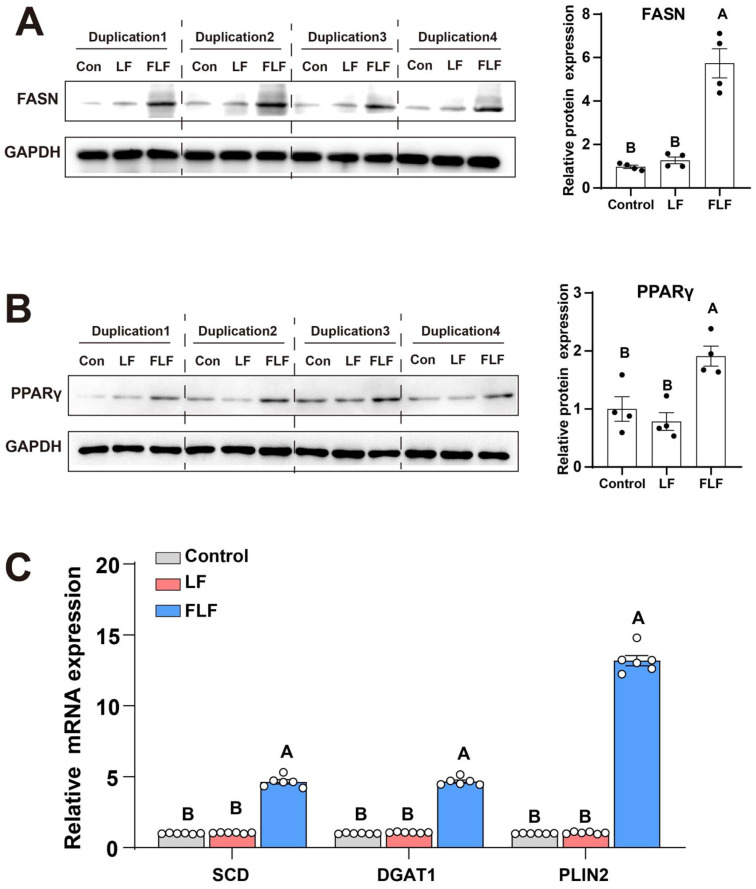
Protein and transcript abundance of components associated with the PPARγ-FASN lipogenic axis in LT muscle. (**A**) Relative FASN protein abundance. (**B**) Relative PPARγ protein abundance. (**C**) Relative mRNA abundance of SCD, DGAT1, and PLIN2. Data are presented as means ± SEM. Western blotting used four biological replicates per treatment; RT-qPCR used six biological replicates per treatment, with three technical replicates per biological sample. Different letters indicate significant differences among dietary treatments (*p* < 0.05).

To examine whether the PPARγ-FASN axis was associated with the lipid-deposition response observed in the FLF group, the PPARγ and FASN protein abundance was quantified. FLF increased the abundance of both proteins relative to CON and LF (Figure 3A,B). RT-qPCR showed higher mRNA abundance of SCD, DGAT1, and PLIN2 in FLF than in CON and LF (Figure 3C). No significant differences were detected between CON and LF (Figure 3C). These findings are consistent with the potential involvement of the PPARγ-FASN axis in the response to the FLF regimen.

## 4. Discussion

Intramuscular fat (IMF) is an important determinant of pork eating quality because it contributes to tenderness, juiciness, and flavor [14]. Previous studies have shown that IMF deposition can be enhanced through nutritional regulation [15,16]. In our study, FLF increased IMF content in the LT muscle, whereas no significant changes were observed in most conventional meat-quality traits. Consistent with our findings, supplementation with 15% or 30% FLF has been reported to increase IMF content in DLY crossbred pigs [7]. However, full-FLF feeding resulted in only a numerical increase in Yuedong Black pigs [6]. This difference may reflect genetic background, baseline IMF content, FLF inclusion level and formulation, fermentation conditions, basal diet composition, slaughter weight, or other study-specific factors. Breed may contribute, but the available cross-study evidence does not establish a breed-dependent response. FLF also increased EAA, lysine, and leucine on a dry-matter basis. Because the crude protein concentration was unchanged, these findings are better interpreted as small shifts in muscle amino-acid composition than as evidence of greater muscle-protein deposition. Protein synthesis, turnover, and amino-acid flux were not measured, and the physiological relevance of these changes should therefore be interpreted cautiously. Fatty-acid profiling further showed that FLF increased several major fatty acids in LT muscle, particularly C16:0, C18:0, C18:1n9, and C18:2n6. The relative increase in summed fatty-acid concentrations was greater than the increase in gravimetric IMF. Gravimetric IMF represents total solvent-extractable lipid, whereas gas chromatography quantifies individual fatty acids after extraction, saponification, methylation, internal-standard correction, and molecular-weight conversion. Both results were expressed on a fresh-tissue wet-weight basis, but IMF was measured in eight pigs per treatment and fatty acids in six. Their relative changes are therefore not expected to be numerically identical. The increase in C16:0 is consistent with greater de novo lipogenesis, although lipogenic flux was not measured.

Further metabolomic profiling revealed that differential metabolites between the FLF group and the CON or LF group were mainly enriched in lipid metabolism. The phospholipid changes were contrast-specific: PS species were displayed for the FLF-versus-CON comparison, whereas PE species were displayed for the FLF-versus-LF comparison. Thus, the present data do not establish that both lipid classes were consistently altered in both comparisons. FLF contains microorganisms and fermentation products [17], microbial membranes contain phospholipids [18], and dietary phospholipids can influence tissue lipid composition [19]. In addition, fermentation-derived metabolites related to methionine and choline metabolism may influence phospholipid synthesis [20]. PS and PE have also been investigated in relation to neural function [21,22], but the present study did not evaluate the functional effects of pork consumption. These possibilities remain hypotheses because dietary phospholipid intake, absorption, and flux into muscle were not directly measured.

Changes in phospholipid composition have been linked to lipid-droplet formation and lipid-metabolic regulation, particularly through phosphatidylcholine, PE, and PS [23]. Proteomic profiling showed that proteins exhibiting differential abundance were predominantly associated with lipid-metabolic processes. FASN and SORBS1 were more abundant in FLF, whereas ACADM, CPT2, and HSD17B4 showed comparison-specific reductions. Correlation analysis indicated positive associations of the displayed PS/PE species with proteins involved in fatty-acid synthesis and negative associations with proteins involved in fatty-acid oxidation. These associations are consistent with coordinated lipid-metabolic remodeling.

Further analysis indicated that the differentially abundant proteins were associated with the PPAR signaling pathway, and transcription-factor prediction identified the PPARγ regulatory network. PPARγ is an important transcriptional regulator of adipogenesis and lipid synthesis [24]. Protein-protein interaction analysis identified FASN, an enzyme involved in de novo fatty-acid synthesis, as a central node [25]. Together, these results suggest the potential involvement of the PPARγ-FASN axis in the lipid-deposition response to FLF. The higher abundance of PPARγ and FASN proteins and the increased mRNA abundance of SCD, DGAT1, and PLIN2 support this interpretation [26,27].

Several limitations should be considered. First, the FLF treatment combined a fermented feed fraction with a microbial-enzyme preparation; consequently, the effects of the microbial inoculum, exogenous enzymes, fermentation-derived metabolites, and their interactions could not be separated. Second, although our findings suggest the involvement of the PPARγ-FASN axis in FLF-induced IMF deposition, we did not systematically investigate whether this pathway represents the key regulatory mechanism underlying the beneficial effects of FLF on IMF accumulation. Future factorial experiments and functional pathway studies are needed to address these limitations.

## 5. Conclusions

In conclusion, the FLF regimen increased IMF content and reduced average backfat thickness in finishing pigs. Integrated metabolomic and proteomic analyses indicated that these changes were associated with alterations in lipid metabolism, including contrast-specific changes in PS and PE species and the potential involvement of the PPARγ-FASN axis. These findings provide evidence that fermented liquid feeding may serve as a nutritional strategy to improve pork quality by modulating muscle lipid deposition.

## Figures and Tables

**Table 2:** Primer sequences used for real-time quantitative PCR.

Genes	Sequence	GenBank Accession no.	Product Size (bp)
*GAPDH*	F: 5′-ACTCACTCTTCTACCTTTGATGCT-3′	NM_001206359.1	110
	R: 5′-TGTTGCTGTAGCCAAATTCA-3′		
*SCD*	F: 5′-TGCTGACTGGTGATGCTGAC-3′	NM_001243798.1	150
	R: 5′-CCTTGATGAGCTGGTGGTGA-3′		
*DGAT1*	F: 5′-ACTGCAGCCTGTACAACGAG-3′	NM_001001170.1	140
	R: 5′-GGCTTGTCAGGTGATGATGG-3′		
*PLIN2*	F: 5′-TTGCTGCTGGTCGATTTCTT-3′	NM_214200.2	129
	R: 5′-CTTCCCATTCTCAGCCTTGAC-3′		

**Table 3:** Carcass traits of growing-finishing pigs.

Item	Control	LF	FLF	*p* Value
Preslaughter live weight	128.44 ± 1.20 ^b^	128.89 ± 1.01 ^b^	133.67 ± 1.53 ^a^	0.01
Carcass weight (kg)	100.78 ± 1.05 ^b^	101.06 ± 0.67 ^b^	105.11 ± 1.67 ^a^	0.02
Dressing percentage (%)	78.46 ± 0.43	78.41 ± 0.20	78.63 ± 0.27	0.88
Carcass straight length (cm)	91.44 ± 1.00	92.50 ± 1.15	93.89 ± 1.01	0.28
Carcass sloping length (cm)	87.00 ± 1.16	88.62 ± 1.15	89.33 ± 0.23	0.31
First-rib backfat thickness (cm)	4.20 ± 0.27	3.94 ± 0.27	3.58 ± 0.18	0.22
Last-rib backfat thickness (cm)	3.42 ± 0.25 ^a^	2.91 ± 0.12 ^ab^	2.62 ± 0.16 ^b^	0.02
Last-lumbar backfat thickness (cm)	2.38 ± 0.24 ^a^	1.98 ± 0.16 ^ab^	1.73 ± 0.08 ^b^	0.05
Average backfat thickness (cm)	3.33 ± 0.23 ^a^	2.94 ± 0.17 ^ab^	2.64 ± 0.12 ^b^	0.04
EMA (cm^2^)	58.07 ± 1.24	57.56 ± 0.83	59.32 ± 0.81	0.44

Abbreviations: Control, basal dry-feed control; LF, liquid-feed treatment; FLF, liquid ration containing 70% unfermented liquid feed and 30% fermented liquid feed; EMA, eye muscle area. Values are means ± SEM (*n* = 8 pigs per treatment). Within a row, means lacking a common superscript differ (*p* < 0.05).

**Table 4:** Meat-quality traits of growing-finishing pigs.

Item	Control	LF	FLF	*p* Value
pH _45 min_	6.02 ± 0.07	6.29 ± 0.10	6.18 ± 0.04	0.07
pH _24 h_	5.59 ± 0.03	5.55 ± 0.02	5.55 ± 0.03	0.54
pH _48 h_	5.45 ± 0.06	5.57 ± 0.06	5.48 ± 0.03	0.24
L* _45 min_	44.24 ± 0.69	43.12 ± 0.44	44.35 ± 0.42	0.22
a* _45 min_	4.54 ± 0.24	4.81 ± 0.33	4.22 ± 0.33	0.40
b* _45 min_	3.83 ± 0.20	3.46 ± 0.19	3.72 ± 0.24	0.46
L* _24 h_	50.63 ± 1.06	49.96 ± 0.74	50.42 ± 0.59	0.84
a* _24 h_	6.30 ± 0.36	6.75 ± 0.52	6.00 ± 0.37	0.47
b* _24 h_	6.86 ± 0.33	6.54 ± 0.41	6.13 ± 0.40	0.41
L* _48 h_	44.05 ± 0.83	44.60 ± 0.55	46.21 ± 0.85	0.14
a* _48 h_	10.21 ± 0.57	10.62 ± 0.18	10.53 ± 0.27	0.73
b* _48 h_	8.32 ± 0.69	7.94 ± 0.33	8.24 ± 0.36	0.85
Shear force (kg)	4.95 ± 0.09	4.81 ± 0.13	4.61 ± 0.03	0.06
Drip loss (%)	2.53 ± 0.20	2.92 ± 0.28	2.13 ± 0.27	0.11
Cooking loss (%)	34.58 ± 0.63	32.70 ± 0.63	33.60 ± 0.56	0.06
Marbling score	2.56 ± 0.17	2.52 ± 0.20	2.93 ± 0.30	0.20

Abbreviations: Control, basal dry-feed control; LF, liquid-feed treatment; FLF, liquid ration containing 70% unfermented liquid feed and 30% fermented liquid feed; L*, lightness; a*, redness; b*, yellowness. Values are means ± SEM (*n* = 8 pigs per treatment).

**Table 5:** Chemical composition of the LT muscle in finishing pigs.

Item	Control	LF	FLF	*p* Value
Moisture (%)	72.09 ± 0.27	72.29 ± 0.15	71.87 ± 0.13	0.33
Crude protein (%)	22.71 ± 0.32	22.77 ± 0.15	23.12 ± 0.15	0.39
Fat (%)	3.07 ± 0.02 ^b^	3.12 ± 0.05 ^b^	3.23 ± 0.04 ^a^	0.02
TG (mmol/g prot)	0.11 ± 0.01 ^b^	0.12 ± 0.01 ^b^	0.17 ± 0.01 ^a^	<0.01
T-CHO (mmol/g prot)	0.18 ± 0.00	0.18 ± 0.009	0.18 ± 0.004	0.82
HDL-C (mmol/g prot)	0.07 ± 0.01	0.07 ± 0.00	0.08 ± 0.01	0.59
LDL-C (mmol/g prot)	0.23 ± 0.01	0.22 ± 0.00	0.23 ± 0.01	0.44

Abbreviations: Control, basal dry-feed control; LF, liquid-feed treatment; FLF, liquid ration containing 70% unfermented liquid feed and 30% fermented liquid feed. Values are means ± SEM (*n* = 8 pigs per treatment). Within a row, means lacking a common superscript differ (*p* < 0.05).

**Table 6:** Amino-acid composition of growing-finishing pigs (% of dry muscle weight).

Item	Control	LF	FLF	*p* Value
EAA ^a^	37.39 ± 0.48 ^b^	38.36 ± 0.31 ^ab^	38.85 ± 0.31 ^a^	0.04
Lysine	6.74 ± 0.10 ^b^	6.86 ± 0.07 ^ab^	7.09 ± 0.06 ^a^	0.02
Phenylalanine	2.94 ± 0.05 ^b^	3.11 ± 0.04 ^a^	3.10 ± 0.04 ^a^	0.03
Methionine	2.05 ± 0.03	2.15 ± 0.03	2.11 ± 0.04	0.12
Threonine	3.56 ± 0.05	3.65 ± 0.04	3.63 ± 0.03	0.31
Valine	3.81 ± 0.04	3.91 ± 0.06	3.94 ± 0.03	0.12
Leucine	6.19 ± 0.10 ^b^	6.34 ± 0.08 ^b^	6.63 ± 0.08 ^a^	0.01
Isoleucine	3.58 ± 0.05	3.61 ± 0.05	3.67 ± 0.05	0.46
Arginine	4.88 ± 0.05	5.01 ± 0.07	4.99 ± 0.07	0.28
Histidine	3.64 ± 0.10	3.73 ± 0.05	3.69 ± 0.09	0.78
NEAA ^b^	35.86 ± 0.49	36.74 ± 0.31	36.77 ± 0.31	0.14
Aspartic acid	7.22 ± 0.11	7.27 ± 0.07	7.33 ± 0.05	0.62
Glutamic acid	11.76 ± 0.22	11.92 ± 0.13	11.94 ± 0.14	0.71
Glycine	3.36 ± 0.03	3.45 ± 0.04	3.40 ± 0.04	0.24
Alanine	4.45 ± 0.05 ^b^	4.65 ± 0.07 ^a^	4.63 ± 0.04 ^a^	0.03
Proline	2.74 ± 0.06	2.82 ± 0.04	2.80 ± 0.03	0.35
Serine	3.08 ± 0.05 ^b^	3.22 ± 0.04 ^a^	3.20 ± 0.02 ^a^	0.04
Tyrosine	2.99 ± 0.08	3.16 ± 0.12	3.20 ± 0.12	0.37
Cysteine	0.26 ± 0.01	0.25 ± 0.01	0.27 ± 0.01	0.38
Total amino acids	73.25 ± 0.94 ^b^	75.10 ± 0.56 ^b^	75.62 ± 0.41 ^a^	0.04
EAA/NEAA	1.043 ± 0.006	1.044 ± 0.003	1.057 ± 0.011	0.33

Abbreviations: Control, basal dry-feed control; LF, liquid-feed treatment; FLF, liquid ration containing 70% unfermented liquid feed and 30% fermented liquid feed; EAA, essential amino acids; NEAA, nonessential amino acids. Values are means ± SEM (*n* = 6 pigs per treatment); within a row, means lacking a common superscript differ (*p* < 0.05). EAA = Lys + Met + Thr + Val + Leu + Ile + Phe + Arg + His; NEAA = Asp + Glu + Ala + Pro + Ser + Cys + Tyr + Gly.

**Table 7:** Fatty-acid concentrations in the longissimus thoracis muscle of finishing pigs, expressed on a fresh-tissue wet-weight basis (mg/100 g fresh muscle).

Item	Control	LF	FLF	*p* Value
SFA ^a^	734.68 ± 4.62	761.98 ± 10.43	926.77 ± 9.08	<0.001
C10:0	2.56 ± 0.11 ^b^	3.36 ± 0.15 ^a^	3.47 ± 0.31 ^a^	0.02
C12:0	2.47 ± 0.63	2.87 ± 0.38	2.73 ± 0.21	0.82
C14:0	25.53 ± 0.38 ^b^	26.41 ± 1.20 ^b^	29.48 ± 0.87 ^a^	0.02
C16:0	441.69 ± 2.26 ^c^	471.39 ± 7.24 ^b^	587.37 ± 7.53 ^a^	<0.001
C18:0	218.18 ± 4.37 ^b^	217.89 ± 3.28 ^b^	267.26 ± 9.45 ^a^	<0.001
C20:0	4.95 ± 0.39	6.03 ± 0.83	7.60 ± 0.92	0.07
MUFA ^b^	748.41 ± 12.83 ^b^	771.50 ± 24.93 ^b^	966.24 ± 12.72 ^a^	<0.001
C16:1	58.62 ± 1.35	60.46 ± 3.11	67.83 ± 3.67	0.09
C18:1n9	675.75 ± 12.35 ^b^	695.78 ± 22.95 ^b^	881.25 ± 14.17 ^a^	<0.001
C20:1n9	10.85 ± 0.54 ^b^	11.72 ± 0.46 ^ab^	13.31 ± 0.67 ^a^	0.02
PUFA ^c^	194.62 ± 7.77 ^b^	207.84 ± 14.18 ^b^	244.58 ± 10.59 ^a^	0.02
C18:2n6	164.32 ± 6.93 ^b^	174.86 ± 11.92 ^b^	208.01 ± 8.09 ^a^	0.01
C18:3n6	1.20 ± 0.05	1.57 ± 0.13	1.70 ± 0.16	0.15
C18:3n3	9.44 ± 0.47 ^b^	11.29 ± 0.85 ^ab^	12.81 ± 0.48 ^a^	0.03
C20:2	6.46 ± 0.25 ^b^	6.90 ± 0.46 ^b^	8.32 ± 0.24 ^a^	<0.01
C20:3n6	2.79 ± 0.21	3.39 ± 0.53	3.64 ± 0.41	0.34
C20:5n3	1.40 ± 0.20	2.12 ± 0.49	2.14 ± 0.49	0.38
C22:6n3	5.68 ± 0.92	4.97 ± 0.85	4.75 ± 0.95	0.75
n-3 PUFA ^d^	15.19 ± 0.79	15.69 ± 1.49	17.15 ± 1.83	0.62
n-6 PUFA ^e^	168.32 ± 7.05 ^b^	179.82 ± 12.39 ^b^	213.34 ± 8.50 ^a^	0.01
n-6/n-3	11.16 ± 0.44	11.63 ± 0.57	12.98 ± 1.03	0.22
PUFA/SFA	0.26 ± 0.01	0.27 ± 0.02	0.26 ± 0.01	0.87

Abbreviations: Control, basal dry-feed control; LF, liquid-feed treatment; FLF, liquid ration containing 70% unfermented liquid feed and 30% fermented liquid feed; SFA, saturated fatty acids; MUFA, monounsaturated fatty acids; PUFA, polyunsaturated fatty acids. Values are means ± SEM (*n* = 6 pigs per treatment); within a row, means lacking a common superscript differ (*p* < 0.05). Totals were calculated from all quantified fatty acids, including minor species not listed individually.

## Data Availability

The original contributions presented in this study are included in the article/Supplementary Materials. Further inquiries can be directed to the corresponding author.

## Supplementary Materials

The following supporting information can be downloaded at: https://www.mdpi.com/article/10.3390/foods15152725/s1, Table S1: Complete fatty-acid profile of the *longissimus thoracis* muscle in finishing pigs fed dry, liquid, or fermented liquid diets.
