# Supplementary material for: Fermented Liquid Feeding Modulates Intramuscular Fat Deposition and Lipid Composition in Pork: Integrated Metabolomic and Proteomic Insights
Source: Foods. 2026 Aug 3;15(15):2725. doi: 10.3390/foods15152725 (PMC13465068; doi:10.3390/foods15152725)
Supplement: Supplementary file 1 [file foods-15-02725-s001.zip › foods-4422638-supplementary.pdf]

Table S1. Complete fatty-acid profile of the *longissimus thoracis* muscle in finishing pigs fed dry, liquid, or fermented liquid diets

Item	Control	LF	FLF	P value
C10:0	2.56 ± 0.11[b]	3.36 ± 0.15[a]	3.47 ± 0.31[a]	0.02
C12:0	2.47 ± 0.63	2.87 ± 0.38	2.73 ± 0.21	0.82
C13:0	0.30 ± 0.04	0.32 ± 0.04	0.34 ± 0.05	0.81
C14:0	25.53 ± 0.38[b]	26.41 ± 1.20[b]	29.48 ± 0.87[a]	0.02
C15:0	3.00 ± 0.25	3.10 ± 0.23	3.20 ± 0.26	0.85
C16:0	441.69 ± 2.26[c]	471.39 ± 7.24[b]	587.37 ± 7.53[a]	<0.001
C17:0	20.00 ± 3.00	16.00 ± 2.80	12.00 ± 2.50	0.16
C18:0	218.18 ± 4.37[b]	217.89 ± 3.28[b]	267.26 ± 9.45[a]	<0.001
C20:0	4.95 ± 0.39	6.03 ± 0.83	7.60 ± 0.92	0.07
C21:0	2.00 ± 0.25	2.00 ± 0.22	2.00 ± 0.24	1.00
C22:0	6.00 ± 1.00	5.50 ± 0.90	5.00 ± 0.80	0.74
C23:0	3.00 ± 0.55	3.00 ± 0.50	3.00 ± 0.48	1.00
C24:0	5.00 ± 1.10	4.11 ± 0.90	3.32 ± 0.80	0.47
**ΣSFA**	**734.68 ± 4.62[b]**	**761.98 ± 10.43[b]**	**926.77 ± 9.08[a]**	**<0.001**
C14:1	0.35 ± 0.07	0.38 ± 0.08	0.42 ± 0.09	0.83
C15:1	0.15 ± 0.04	0.16 ± 0.04	0.18 ± 0.05	0.89
C16:1	58.62 ± 1.35	60.46 ± 3.11	67.83 ± 3.67	0.09
C17:1	1.15 ± 0.20	1.25 ± 0.21	1.35 ± 0.22	0.80
C18:1n9	675.75 ± 12.35[b]	695.78 ± 22.95[b]	881.25 ± 14.17[a]	<0.001
C20:1n9	10.85 ± 0.54[b]	11.72 ± 0.46[ab]	13.31 ± 0.67[a]	0.02
C22:1n9	0.75 ± 0.12	0.82 ± 0.13	0.88 ± 0.14	0.78
C24:1n9	0.79 ± 0.18	0.93 ± 0.20	1.02 ± 0.21	0.71
**ΣMUFA**	**748.41 ± 12.83[b]**	**771.50 ± 24.93[b]**	**966.24 ± 12.72[a]**	**<0.001**
C18:2n6	164.32 ± 6.93[b]	174.86 ± 11.92[b]	208.01 ± 8.09[a]	0.01
C18:3n6	1.20 ± 0.05	1.57 ± 0.13	1.70 ± 0.16	0.15
C18:3n3	9.44 ± 0.47[b]	11.29 ± 0.85[ab]	12.81 ± 0.48[a]	0.03
C20:2	6.46 ± 0.25[b]	6.90 ± 0.46[b]	8.32 ± 0.24[a]	<0.01
C20:3n3	0.45 ± 0.10	0.50 ± 0.12	0.60 ± 0.14	0.68
C20:3n6	2.79 ± 0.21	3.39 ± 0.53	3.64 ± 0.41	0.34
C20:4n6	2.30 ± 0.40	1.65 ± 0.35	1.95 ± 0.38	0.49
C20:5n3	1.40 ± 0.20	2.12 ± 0.49	2.14 ± 0.49	0.38
C22:2	0.58 ± 0.12	0.59 ± 0.11	0.66 ± 0.13	0.88
C22:6n3	5.68 ± 0.92	4.97 ± 0.85	4.75 ± 0.95	0.75
**ΣPUFA**	**194.62 ± 7.77[b]**	**207.84 ± 14.18[b]**	**244.58 ± 10.59[a]**	**0.02**
**n-3 PUFA**	**16.97 ± 1.06**	**18.88 ± 1.30**	**20.30 ± 1.19**	**0.17**
**n-6 PUFA**	**170.61 ± 6.95[b]**	**181.47 ± 11.94[b]**	**215.30 ± 8.13[a]**	**0.01**
**n-6/n-3**	**10.05 ± 0.64**	**9.61 ± 0.72**	**10.61 ± 0.76**	**0.62**
**PUFA/SFA**	**0.26 ± 0.01**	**0.27 ± 0.02**	**0.26 ± 0.01**	**0.87**

Data are presented as means ± SEM (n = 6 biological replicates per treatment) and expressed as mg/100 g fresh muscle on a wet-weight basis. Different superscript letters within a row indicate significant differences among dietary treatments ($P < 0.05$). CON, basal dry-feed control; LF, liquid-feed treatment; FLF, liquid ration containing 70% unfermented liquid feed and 30% fermented liquid feed; SFA, saturated fatty acids; MUFA, monounsaturated fatty acids; PUFA, polyunsaturated fatty acids.

ΣSFA = C10:0 + C12:0 + C13:0 + C14:0 + C15:0 + C16:0 + C17:0 + C18:0 + C20:0 + C21:0 + C22:0 + C23:0 + C24:0. ΣMUFA = C14:1 + C15:1 + C16:1 + C17:1 + C18:1n9 + C20:1n9 + C22:1n9 + C24:1n9. ΣPUFA = C18:2n6 + C18:3n6 + C18:3n3 + C20:2 + C20:3n3 + C20:3n6 + C20:4n6 + C20:5n3 + C22:2 + C22:6n3. n-3 PUFA = C18:3n3 + C20:3n3 + C20:5n3 + C22:6n3. n-6 PUFA = C18:2n6 + C18:3n6 + C20:3n6 + C20:4n6. Triundecanoin was used as the internal standard (4.00 mg/mL; 1.00 mL added to each fresh-muscle sample before lipid extraction).
